# Real-world effectiveness of vedolizumab in refractory pouchitis: a focus on biomarker changes during intravenous to subcutaneous transition (POUCHIVED study)

**DOI:** 10.3389/fimmu.2025.1630678

**Published:** 2025-12-17

**Authors:** Leticia Gimeno-Pitarch, Elena Céspedes-Martínez, Rocío Ferreiro-Iglesias, María Dolores Martín-Arranz, Beatriz Sicilia, Inma Lorca, José Huguet, Francisco Mesonero, Raquel Vicente-Lidón, Alejandro Mínguez, Pablo Galvez-Martín, Xavier Serra-Ruiz, Manuel Barreiro-de Acosta, Pilar Nos, Marisa Iborra

**Affiliations:** 1 Gastroenterology Department, Hospital General Universitario de Castellón, Castellón, Spain; 2 Unitat d’Atenció Crohn-Colitis (UACC), Hospital Universitario Vall d’Hebron, Barcelona, Spain; 3 Gastroenterology Department, Hospital Clínico Universitario de Santiago, Santiago de Compostela, Spain; 4 Gastroenterology Department, Hospital Universitario La Paz de Madrid, Madrid, Spain; 5 Gastroenterology Department, Hospital Universitario de Burgos, Burgos, Spain; 6 Gastroenterology Department, Hospital Clínico Universitario de Valencia, Valencia, Spain; 7 Gastroenterology Department, Hospital General Universitario de Valencia, Valencia, Spain; 8 Gastroenterology Department, Hospital Universitario Ramón y Cajal de Madrid, Madrid, Spain; 9 Gastroenterology Department, Hospital Universitario Miguel Servet de Zaragoza, Zaragoza, Spain; 10 Inflammatory Bowel Disease (IBD) Research Group, Health Research Institute La Fe (IIS La Fe), Valencia, Spain; 11 IBD Unit, Gastroenterology Department, Hospital Universitario la Fe de Valencia, Valencia, Spain; 12 Gastroenterology Department, Hospital Universitario Vall d’Hebron de Barcelona, Barcelona, Spain

**Keywords:** chronic antibiotic-refractory pouchitis (CARP), ileal pouch-anal anastomosis (IPAA), inflammatory bowel disease, ulcerative colitis (UC), vedolizumab

## Abstract

**Background:**

Chronic antibiotic-refractory pouchitis (CARP) is a complex and often debilitating complication following ileal pouch-anal anastomosis (IPAA). Vedolizumab (VDZ), a gut-selective anti-integrin agent, has shown potential efficacy in this context, but real-world evidence remains limited. This study aimed to evaluate the effectiveness, safety, and treatment persistence of VDZ in patients with CARP, including outcomes after transitioning from intravenous (IV) to subcutaneous (SC) administration.

**Methods:**

This multicenter Spanish study (POUCHIVED) included patients with CARP treated with VDZ. Disease activity was assessed using the modified Pouchitis Disease Activity Index (mPDAI). mPDAI, fecal calprotectin (FC), C-reactive protein (CRP), and serum VDZ levels were recorded at weeks 12, 24, and 52 during IV treatment, and subsequently after switching to SC administration when applicable.

**Results:**

Forty-seven patients (66% male; median age 51 years) were included, of whom 53% transitioned to SC VDZ. Most patients presented mild pouchitis at baseline, reflecting early initiation of vedolizumab in clinical practice. IV VDZ therapy led to significant reductions in mPDAI and FC at all evaluated time points: mPDAI decreased from a baseline median of 5 [IQR 3–7] to 3 [2–5], 2 [2–5], and 2 [2–5.8] at weeks 12, 24, and 52, respectively (p<0.05) and FC declined from 443 µg/g [IQR 213–746] at baseline to 329 µg/g [145–701], 193 µg/g [91–412], and 178 µg/g [101–640] at the same intervals. Following the switch to SC VDZ, both mPDAI and FC showed further improvement at week 52, while CRP levels remained unchanged. SC VDZ was associated with higher serum drug concentrations compared to IV. Better clinical outcomes were observed in patients with fewer prior therapies post-surgery, while baseline corticosteroid or immunosuppressant use and prior VDZ exposure before IPAA did not influence response. Treatment persistence was high, and adverse event rates were comparable between IV and SC formulations (8.5% *vs*. 12%).

**Conclusions:**

VDZ is effective and safe for the management of CARP, particularly when used at first line in the treatment course. Transitioning from IV to SC administration maintains clinical remission and is well tolerated in real-world clinical settings.

## Introduction

Despite advances in ulcerative colitis (UC) treatment, approximately 25–30% of patients eventually require a colectomy, being the restorative proctocolectomy and ileal pouch-anal anastomosis (IPAA) the preferred surgical approach (1). This procedure is linked to various inflammatory complications, such as pouchitis, cuffitis and Crohn’s disease (CD) like condition of the pouch, with similar symptoms, which require the combined assessment of clinical, endoscopic and histological features for their diagnosis (1). These complications are common, with 53% developing acute pouchitis, 30% cuffitis, and 12% CD-like condition of the pouch (2).

Pouchitis is the most frequent complication, affecting up to 50% of patients within the first two years of post-surgery. Most cases are mild, self-limited, and respond well to antibiotics, but approximately 17% develop chronic pouch inflammation, categorized as chronic antibiotic-dependent pouchitis (CADP) or chronic antibiotic-refractory pouchitis (CARP) (3). CADP responds to long-term antibiotic therapy, whereas CARP persists despite a four-week course of optimized treatment with antibiotics (4–7).

Acute pouchitis is often associated with bacterial overgrowth due to post-surgical ileal changes, explaining its response to antibiotics (4). In contrast, chronic pouchitis has a largely unknown, probably multifactorial etiology involving immune and microbiome dysregulation in genetically predisposed individuals. This supports the use of budesonide and advanced biological therapies (5–8). Recent guidelines recommend advanced therapies for CADP and CARP once infections or structural causes are excluded (5–10). Previous observational studies supported the use of advanced IBD therapy for the treatment of CARP; although its therapeutic approach continues to be a challenge in clinical practice. Recent retrospective, observational multicenter study shows the difficulty of maintaining CARP under control in long-term follow-up with anti-TNF drugs, classically used as the first-line advanced treatment in these context (7). Moreover, discontinuation of anti- TNF drugs caused reservoir failure, highlighting the need to find valid and persistent therapeutic options. Vedolizumab (VDZ) is recommended as the first-line option on international guidelines (8).

VDZ, a monoclonal antibody against α4β7 integrin, has demonstrated efficacy in CARP with clinical remission rates up to 20%- 25% in UC and CD (11). EARNEST trial is the only study in chronic pouchitis that has shown higher clinical remission rates with VDZ compared to placebo (31% vs. 10% and 35% vs. 18% at weeks 14 and 34) (12). In fact, the results of this double-blind, randomized controlled trial with placebo (1:1 ratio), achieved the formal indication of VDZ for this clinical scenario, along with other small retrospective cohort studies limited by their observational nature, low sample size and short-term follow-up (3, 11, 13–16). Besides, it is worth saying that enrolled patients in EARNEST trial treated with VDZ continued antibiotic therapy during the first month, and even 21% of patients at week 34, which could have influenced the outcomes (12).

In our previous experience, SC VDZ has proven to be an effective therapeutic alternative in patients with IBD, showing higher trough serum levels compared to the IV formulation (17). Moreover, we observed that switching from IV to SC VDZ in patients already under treatment was associated with a high persistence rate at one year, and a trend toward greater biochemical remission—particularly among those previously on intensified IV regimens—without the need for SC intensification (18). Building on this evidence, the present real-world study evaluates the effectiveness and safety of IV VDZ in patients with CARP (POUCHIVED study), as well as clinical and biochemical outcomes following the transition to SC administration. The primary objective was to assess effectiveness based on changes in the modified Pouch Disease Activity Index (mPDAI). Secondary endpoints included the identification of response predictors, evaluation of biomarkers and serum drug levels before and after switching, and comparison of treatment persistence and safety between IV and SC VDZ formulations.

## Methods

This observational real-life multicenter study was conducted in 10 Spanish hospitals. Data on patients receiving VDZ for chronic pouchitis were consecutively collected by IBD expert’s groups using a self-administered Excel spreadsheet.

### Patients

All included patients had UC and had undergone IPAA due to the absence of therapeutic medical options. Subsequently, they had been diagnosed with CARP because of persistent symptoms of active pouchitis despite several antibiotic treatments and an endoscopic evaluation of the pouch which ruled out infection or other complications.

This was a mixed cohort study: patients with a diagnosis of chronic antibiotic-refractory pouchitis treated with vedolizumab were prospectively followed for a minimum of 52 weeks. Additionally, retrospective chart review provided information on prior therapies and baseline demographics. Ten hospitals participated and enrolment began in early 2020 with consecutive inclusion of eligible cases until 2024.

To ensure data quality, all centers applied uniform definitions of CARP and inclusion/exclusion criteria. Variables were predefined and entered into a standardized Excel database to avoid heterogeneity in coding. Key outcomes were collected according to unified protocols and centrally reviewed. Two independent reviewers performed systematic data cleaning prior to analysis. Furthermore, all participating investigators were IBD specialists from referral centers, which minimized diagnostic variability and increased dataset reliability.

All patients were aged 18 years or older at enrolment and had chronic refractory pouchitis. Exclusion criteria included CD of the pouch, CADP unless all episodes were refractory, and failure to complete the full VDZ induction regimen.

All patients received IV VDZ at the standard dosing regimen for UC: 300 mg at weeks 0, 2 and 6, followed by 300 mg every 8 weeks. The dose was increased to 300 mg every 6 or 4 weeks when the patient experienced a loss of response. The switch to SC VDZ (108 mg every 2 weeks) was performed on the same day as the scheduled IV infusion.

Concomitant therapies during follow-up were recorded, including corticosteroids and immunosuppressants (azathioprine, mercaptopurine or methotrexate).

### Demographic and clinical variables

Demographic and clinical data were collected, including date of birth, sex, smoking status, age at UC onset, disease extent at diagnosis and the presence of extra-intestinal manifestations (EIMs). Information on advanced therapies used before and after surgery—prior to initiating IV VDZ—were also gathered, including exposure to anti-TNFα agents, VDZ, ustekinumab, and JAK inhibitors. Additionally, the use of concomitant corticosteroids and/or immunosuppressants (azathioprine, mercaptopurine, methotrexate), or their initiation during VDZ treatment after IPAA, was also recorded.

Clinical outcomes were assessed using the mPDAI and biomarkers such as fecal calprotectin (FC) and C-reactive protein (CRP). We also recorded VDZ through serum levels measured on the day of drug administration. They were recorded at baseline, week 12, 24 and 52 after the start of IV VDZ. The intensification of IV VDZ treatment prior to the switch was also documented.

To assess the safety of VDZ treatment in CARP, adverse events (AEs) and hospitalizations, including surgery for permanent ileostomy, were documented during the follow-up period. In addition, maintenance of IV and SC VDZ was recorded to determine the persistence of the treatment, noting the reasons for its discontinuation.

### Definitions

The mPDAI used in this study included clinical symptoms (stool frequency, rectal bleeding, urgency/abdominal pain, incontinence, fever; range 0–6) and endoscopic features (erythema, friability, edema, erosions/ulcers; range 0–6), without histology. Scores <5 indicate quiescent pouchitis, 5–8 moderate and 9–12 severe disease, according to EARNEST trial (12). Some patients initiated VDZ early as first-line post-surgery therapy to prevent recurrence and were therefore in clinical remission at treatment start (mPDAI <5). Similarly, a FC level of ≤250 μg/g was considered within the normal range (12).

Clinical remission was defined as a mPDAI score of less than 5 and combined clinical and biochemical remission was defined as mPDAI <5 and FC ≤250 µg/g, representing a more stringent therapeutic goal.

Persistence was defined as the time from treatment initiation to discontinuation for any reason (primary failure, loss of response, adverse event or withdrawal), using all patients initiating IV or SC vedolizumab as denominators.

### Statistical analysis

Quantitative variables were reported as mean ± SD or median (IQR), and qualitative ones as percentages. Data over time (mPDAI, FC, CRP) were shown using box plots. Normality was assessed with the Kolmogorov-Smirnov test; non-parametric tests were used when p<0.05.

All analyses were performed according to the intention-to-treat principle, including all patients initially enrolled regardless of loss to follow-up or treatment discontinuation. Paired comparisons at baseline and weeks 12, 24, and 52 were made using Wilcoxon or repeated measures t-tests, depending on data distribution. Univariable analyses (Mann-Whitney U or t-tests) examined associations with treatments before/after surgery, antibiotic use, and combination therapy. Multivariable linear regression was planned for factors affecting mPDAI, FC, and CRP over time.

Drug persistence (IV vs. SC) was assessed with Kaplan-Meier curves and compared using the log-rank test. Significance was set at p<0.05. Analyses were done with IBM SPSS.

## Results

### Patient demographics and disease characteristics

A total of 47 patients with IPAA receiving IV VDZ for chronic pouchitis were included. All patients were followed from the start of IV therapy. Based on routine clinical practice, all were offered a switch to SC administration; 25 patients (53%) accepted and were subsequently followed.

The demographic and clinical characteristics of the population are shown in **Table 1**. Thirty-one patients (66%) were male and the median age at diagnosis of UC was 34 years (20, 41). The majority (85%) had extensive preoperative colonic involvement (E3 of Montreal Classification) and the median time to proctocolectomy with IPAA was 4.7 years.

**Table 1:** Demographic characteristics and use of advanced therapies before and after surgery are reported for the entire cohort (n=47), as well as separately for patients treated with intravenous vedolizumab (IV vedolizumab non switchers; n=22) and patients who switched to subcutaneous vedolizumab (IV vedolizumab switchers; n=25). Data are expressed as median (interquartile range) or number (%).

	All cohort (N = 47)	IV vedolizumab non-switchers (N = 22)	IV vedolizumab switchers (N = 25)
Sex (Male/Female)	31(66%)/16(34%)	15(68%)/7(32%)	16(64%)/9(36%)
Age UC diagnosis (years)	34 [20, 41]	33 [25, 40]	23 [19, 42]
Smoker			
No	34 (55.3%)	16 (72%)	18 (72%)
Yes	6 (12.8%)	4 (18.2%)	2 (8%)
Ex	7 (14.9%)	2 (9%)	5 (2%)
Disease location			
E1	0 (0%)	0 (0%)	0 (0%)
E2	7 (15%)	4 (18%)	3 (12%)
E3	40 (85%)	18 (82%)	22 (88%)
EIM	9 (19%)	4 (18%)	5 (20%)
Biologic pre-surgery	24 (51%)	11 (50%)	13 (52%)
Anti-TNF	24 (51%)	11 (50%)	13 (52%)
Vedolizumab	7 (15%)	3 (14%)	4 (16%)
Ustekinumab	4 (8.5%)	2 (9%)	2 (8%)
JAK inhibitors	3 (6%)	2 (9%)	1 (4%)
Age at surgery (years)	38.3 [28.6, 47.2]	40.6 [32.5, 47.8]	38.3 [24.3, 43.9]
Post-surgery antibiotics	44 (93.6%)	20 (90.9%)	24 (96%)
Post-surgery immunosuppressant	7 (14.9%)	3 (13.6%)	4 (16%)
Biologic post-surgery	18 (38.3%)	13 (59.1%)	5 (20%)
Anti-TNF	17 (36.1%)	12 (54.5%)	5 (20%)
Ustekinumab	10 (21.3%)	8 (36.4%)	2 (8%)
JAK inhibitors	2 (4.3%)	1 (4.5%)	1(4%)
Time until start VDZ initiation (years)	51.3 [42.6, 63.5]	49.4 [41.5, 63.9]	53.5 [42.6, 61.2]
Time from CU diagnosis to VDZ initiation (years)	19.5 [11.6, 25.9]	12.1 [11.4, 24.5]	21.9 [13.4, 29]
Corticosteroids at start	14 (29.8%)	6 (27.3%)	8 (32%)
Immunosuppressant at start	6 (12.8%)	2 (9%)	4 (16%)

Ulcerative colitis (UC); serum C-reactive protein (CRP); faecal calprotectin (FC); UC extension: E1: proctitis, E2 center side UC, E3: extensive UC; extraintestinal manifestations (EIM); vedolizumab (VDZ); intravenous (IV); subcutaneous (SC).

A total of 24 (51.1%) had been treated with biological agents prior to IPAA. After surgery, 44 (93.6%) patients received multiple optimized antibiotic regimens without clinical success. In fact, due to antibiotic failure, 7 (14.9%) were treated with immunosuppressants and up to 18 (38.3%) with biologic treatments (anti-TNF, ustekinumab and JAK inhibitors). Three patients discontinued antibiotic therapy due to intolerance or adverse events, precluding further optimized regimens. These cases were classified as antibiotic-refractory in clinical practice and were therefore retained in the analysis. None of the patients were on antibiotics at the time of IV VDZ initiation or received antibiotics during follow-up. Fourteen patients (29.8%) received concomitant corticosteroids at start, but none of them continued corticosteroid therapy beyond the first two months of IV Vedolizumab. Six patients (12.8%) were treated with immunosuppressants in combination with VDZ during follow-up. Twenty patients (42.5%) received an intensified IV regimen (every 4–6 weeks) before switching to SC administration. As intensification was mostly performed shortly before the switch, follow-up was insufficient to allow a separate outcome analysis.

### Effectiveness of intravenous vedolizumab in chronic refractory pouchitis

**Figure 1:**
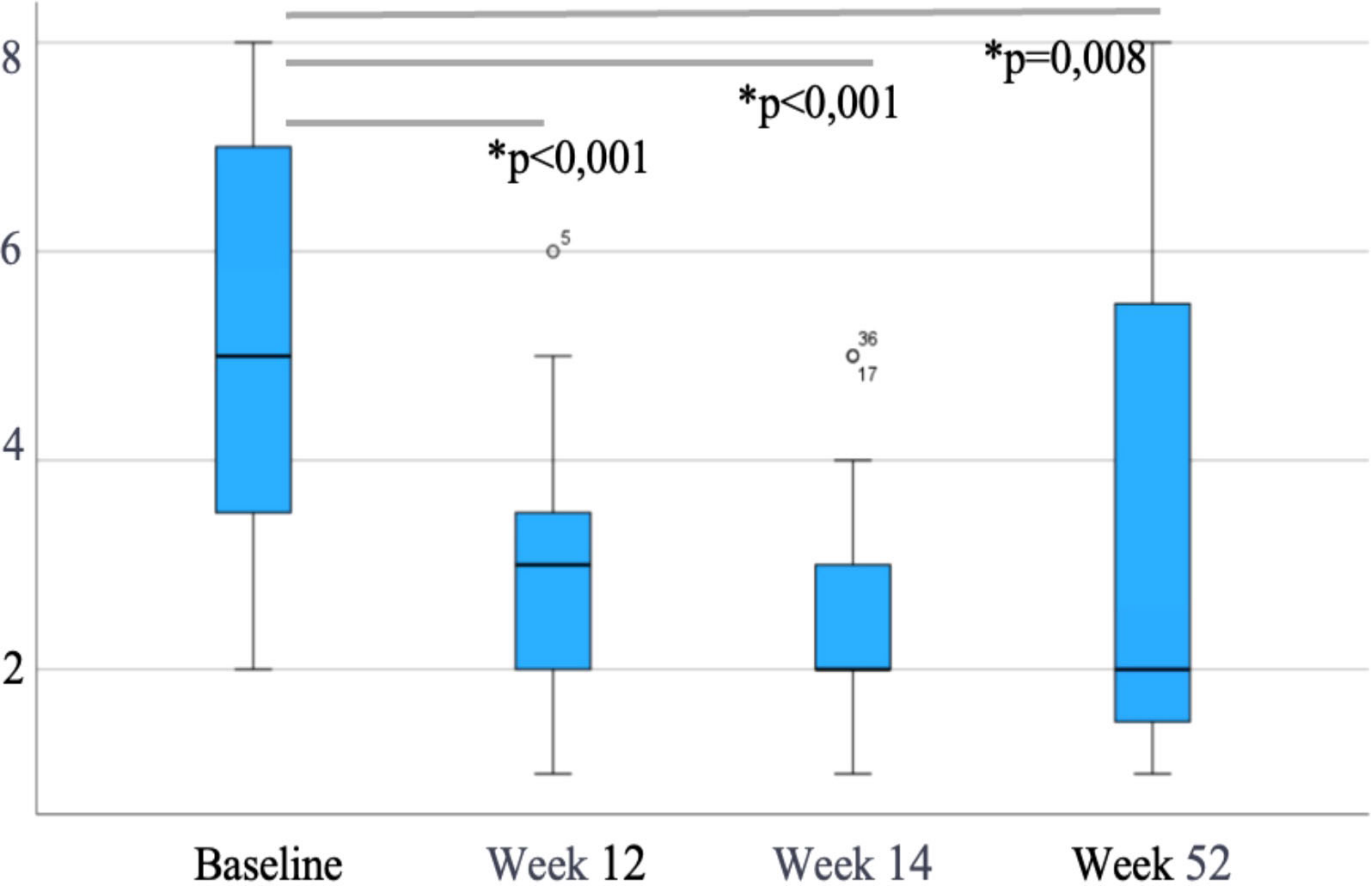
Modified pouchitis disease activity index (mPDAI) levels during follow-up in patients treated with intravenous vedolizumab (n=47). Data are shown as median and interquartile range. *p*-values represent comparisons with baseline (Wilcoxon test). * means "statistically significant".

**Figure 2:**
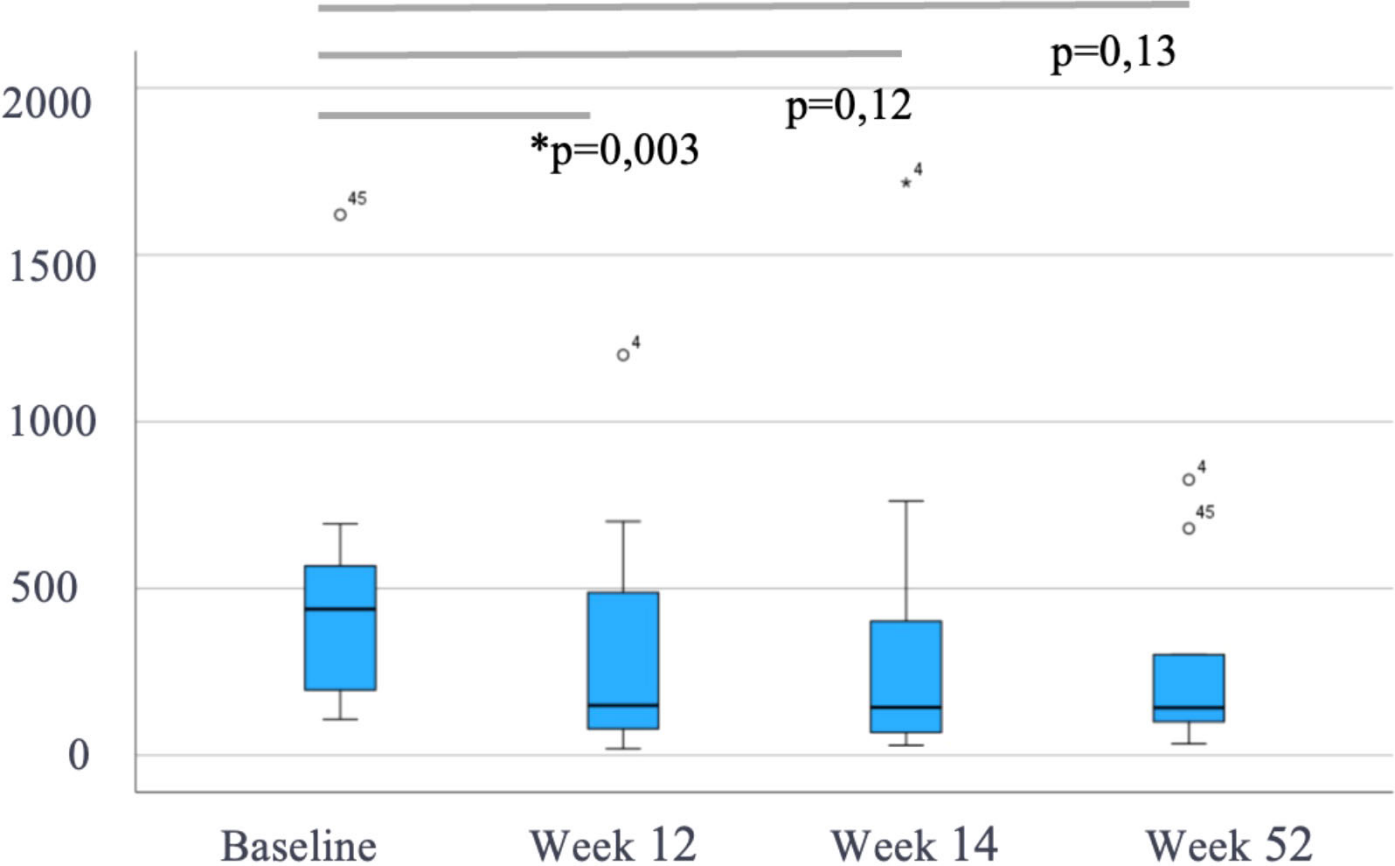
Fecal calprotectin (FC, µg/g) during follow-up in patients treated with intravenous vedolizumab (n=47). Data are shown as median and interquartile range. p-values represent comparisons with baseline (Wilcoxon test). * means "statistically significant".

**Figure 3:**
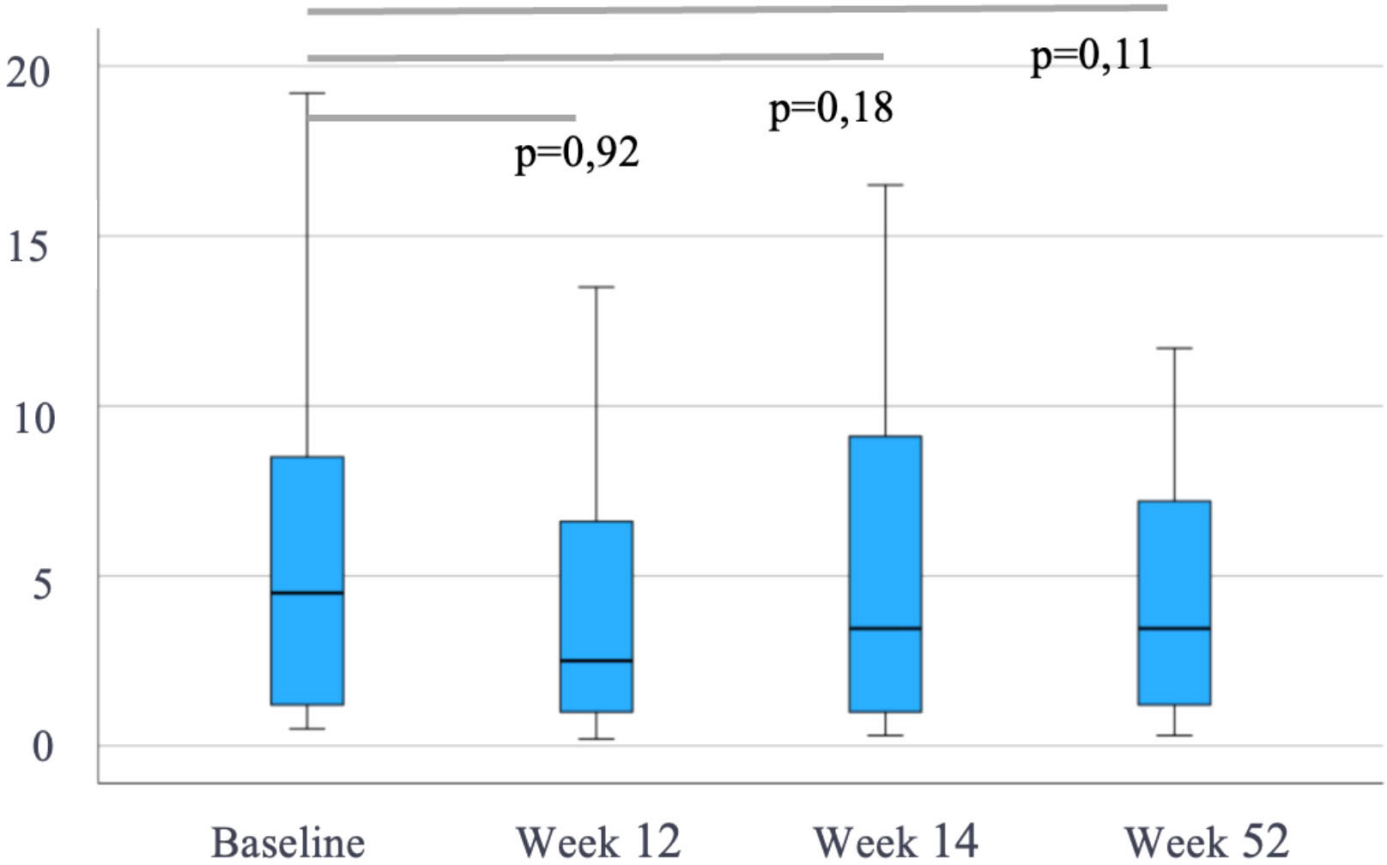
C-reactive protein (CRP, mg/L) during follow-up in patients treated with intravenous vedolizumab (n=47). Data are shown as median and interquartile range. p-values represent comparisons with baseline (Wilcoxon test).

Of the 47 patients starting IV VDZ for CARP, the mean mPDAI at baseline was 5 [3-7] and decreased significantly to 3 [2-5], 2 [2-5] and 2 [2-5.8] at weeks 12, 24 (p<0.001) and 52 (p=0.018), respectively (**Figure 1**). Similarly, FC levels showed a decreasing trend over time from a baseline median of 443 µg/g (IQR 213-746) to 329 µg/g (145-701) at week 12, 193 µg/g (91-412) at week 24 and 178 µg/g (101-640) at week 52. However, only the reduction in week 12 reached statistical significance (p=0.003, p=0.12 and p=0.13 at weeks 12, 24 and 52, respectively) (**Figure 2**). CRP levels remained relatively stable with a median of 4 mg/L [2.3-9.9] at baseline, 4 [1.7-7.2] at week 12, 5.3 [1.8-14.3] at week 24 and 4 [1.4-7.4] at week 52, with no evidence of significant change (p>0.05) (**Figure 3**).

Response rates (mPDAI <5) were as follows: IV group – week 12: 24/41 (58.5%, 95% CI 43.7–72.7); week 24: 19/26 (73.1%, 95% CI 54.6–87.0); week 52: 12/18 (66.7%, 95% CI 44.3–84.3). SC group – week 12: 18/22 (81.8%, 95% CI 62.4–93.6); week 24: 11/12 (91.7%, 95% CI 64.1–97.9); week 52: 6/6 (100%, 95% CI 60.7–99.7). Endoscopic evaluation was available in ~60% of patients at follow-up; a sensitivity analysis restricted to these cases yielded consistent results.

IV VDZ serum levels also showed a downward trend during follow-up, decreasing from a median of 16.9 µg/mL [IQR 10.3-25.7] at baseline to 9 µg/mL [6.7-13.9] at week 52, although this change was not statistically significant (p=0.25) (**Table 2**).

**Table 2:** Median vedolizumab serum concentrations during follow-up in patients receiving intravenous (IV, n=47) and subcutaneous (SC, n=25) formulations.

VDZ-TL	Basal	Week 12	Week 24	Week 52
IV-TL (µg/mL)		16.9 [10.3-25.7]	12 [7.3-23]	9 [6.7-13.9]
SC-TL (µg/mL)	8.5 [7.2-14]	22.7 [20.9-27.3]	19.6 [16.5-21]	21.6 [16.9-26.2]

Intravenous (IV) and subcutaneous (SC) vedolizumab trough level (VDZ-TL).

### Evolution after switching from intravenous to subcutaneous vedolizumab

Twenty-five patients switched to SC VDZ, 18 of them before week 24, with a median duration of 15 weeks (range 14–47) on IV VDZ before the switch. During the first 6 months after transitioning to SC VDZ, no significant changes were observed in mPDAI, fecal calprotectin (FC), or CRP levels. However, by week 52, both mPDAI and FC levels showed a significant reduction compared to pre-switch values (p = 0.04 and p = 0.038, respectively) (**Table 3**).

**Table 3:** Evolution of mPDAI, FC, CRP levels after switching from intravenous to subcutaneous vedolizumab (n=25).

SC VDZ-TL (N = 25)	Baseline	Week 12	Week 24	Week 52
mPDAI	2 [1-4.3]	2 [1-3]	1 [1-2]	1.5 [0.25-2]
FC (µg/g)	550 [1445- 1163]	264 [52-989]	129 [101-448]	62 [31-211]
CRP (mg/L)	4 [3.2-12.5]	4 [2.6-14.8]	4 [1.3-4]	2.7 [1.3-4]
VDZ-TL (µg/mL)	8.5 [7.2-14]	22.7 [20.9-27.3]	19.6 [16.5-2]	21.6 [16.9-26.2]

Modified Pouch Disease Activity Index (mPDAI); serum C-reactive protein (CRP); faecal calprotectin (FC); subcutaneous vedolizumab trough level (SC VDZ-TL).

**Figure 4:**
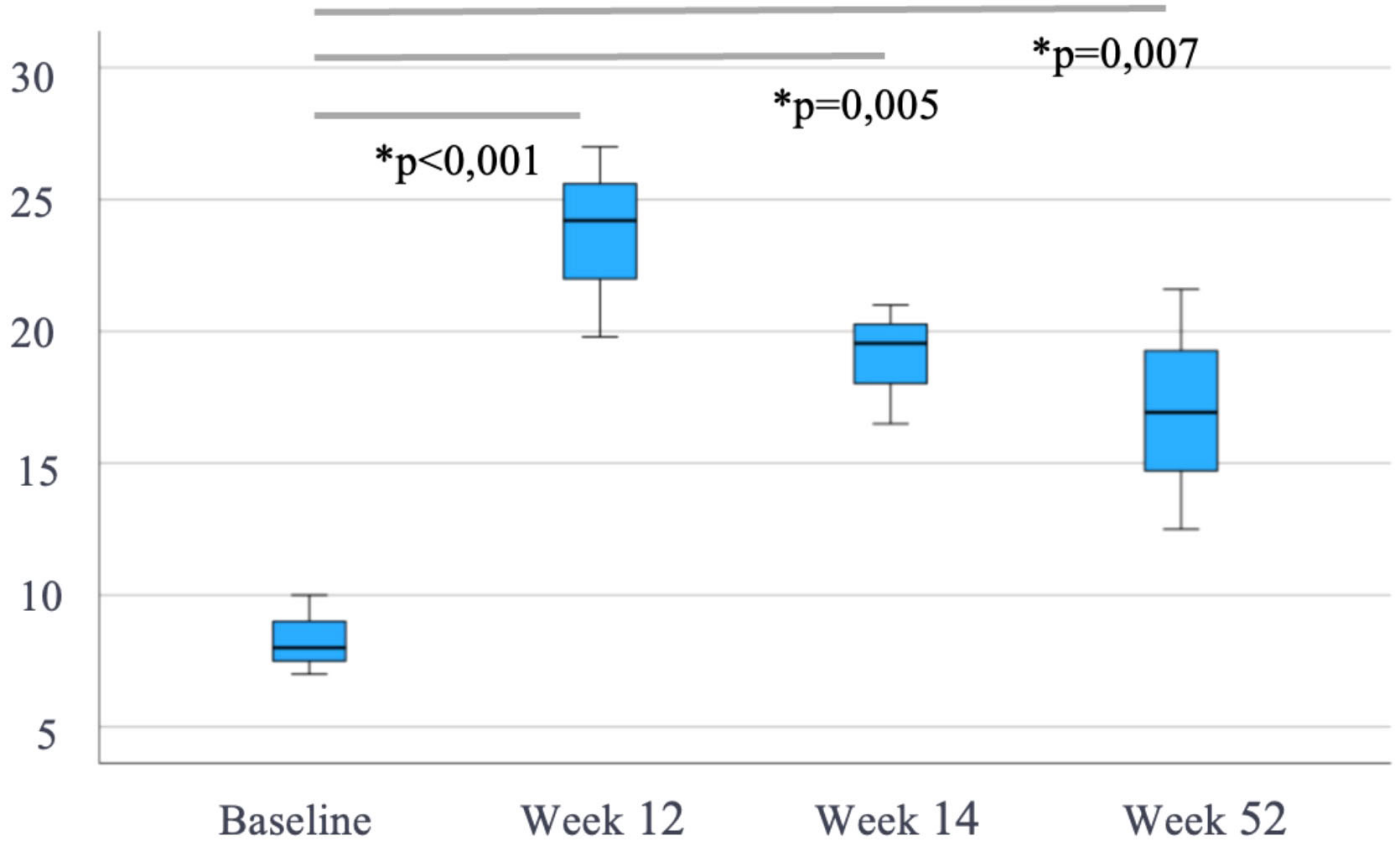
Evolution of subcutaneous vedolizumab trough levels (µg/mL) after switching from intravenous vedolizumab (n=25). Median (IQR), *p*-values *vs* pre-switch levels. * means "statistically significant".

In addition, serum levels of SC VDZ increased significantly over time from a pre-switch median of 8.5 µg/mL [7.2-14] to 22.7 µg/mL [20.9-27.3], 19.6 µg/mL [16.5-21] and 21.6 µg/mL [16.9-26.2] at subsequent time points (p<0.007) (**Table 2**, **Figure 4**).

### Concentration of VDZ levels associated with therapeutic outcomes at different time points

**Table 4** presents trough levels of IV and SC VDZ at different time points and their association with clinical remission and combined clinical and biochemical remission. Due to the limited number of patients in each subgroup, these observations should be interpreted cautiously, as statistical significance could not be assessed.

**Table 4:** Concentration of vedolizumab levels associated with therapeutic outcomes at different time points.

	Intravenous vedolizumab through levels		Subcutaneous vedolizumab through levels	
Week (W)	Clinical remission	Combined remission	Clinical remission	Combined remission
W 12 *YES	21.5 (11.1, 30.7) (n=14)	19.4 (9.2, 22) (n=5)	23 (21.1, 27.4) (n=13)	24.2 (21.5, 27) (n=5)
**NO	17.1 (13.2, 23.8) (n=7)	17.1 (14.8, 21) (n=5)	20.8 (one patient)	20.8 (one patient)
W 24 *YES	10.3 (6.9, 14.5) (n=6)	10.3 (9.5, 11) (n=2)	19.6 (16.5, 21) (n=5)	20.3 (18.9, 22.3) (n=4)
**NO	22.2 (one patient)	None	None	None
W 56 *YES	6.5 (4.4, 9.3)	12 (10.5, 13)	21.6 (16.9, 26.2) (n=5)	21.6 (17.1, 24.8) (n=3)
**NO	9 (one patient)	None	None	None

Clinical remission was defined as a modified Pouch Disease Activity Index (mPDAI) score <5 and combined clinical and combined remission was defined as mPDAI<5 and faecal calprotectin ≤250 µg/g. *Vedolizumab levels in patients who achieved clinical remission with and without normalization faecal calprotectin levels (YES) and **vedolizumab levels in patients who did not achieve clinical remission with and without normalization faecal calprotectin levels (NO) in each evaluated time point. Data are presented as median vedolizumab trough levels with interquartile ranges. Due to the limited number of patients in each subgroup, the statistical significance could not be assessed.

Overall, patients receiving SC VDZ exhibited higher and more stable trough levels compared to those on IV therapy. Additionally, achieving combined clinical and biochemical remission was generally associated with higher drug levels than clinical remission alone.

At week 12, patients who achieved clinical remission had higher VDZ levels compared to those who did not, for both IV and SC routes (IV: 21.5 µg/mL [IQR 11.1–30.7] vs. 17.1 µg/mL [IQR 13.2–23.8]; SC: 23.0 µg/mL [IQR 21.1–27.4] vs. 20.8 µg/mL). When analyzing the more stringent outcome of combined remission, trough levels were slightly lower in the IV group (19.4 µg/mL) compared to those achieving clinical remission alone (21.5 µg/mL), whereas SC levels were higher (24.2 µg/mL vs. 23.0 µg/mL). Moreover, patients who achieved either clinical or combined remission with the IV formulation showed higher VDZ concentrations comparable to those observed with the SC route. This convergence likely reflects two factors: IV samples were drawn right after the induction phase, when serum levels are still elevated, while the SC formulation inherently sustains higher steady-state concentrations.

At week 52, IV-treated patients who achieved clinical remission had lower VDZ trough levels (6.5 µg/mL [IQR 4.4–9.3]) compared to those who achieved the more stringent outcome of combined remission (11.5 µg/mL [11.0–12.0]). However, both groups exhibited markedly lower concentrations than patients treated with the SC formulation. Among the SC group, high and stable trough levels were maintained regardless of the remission type (clinical remission: 21 µg/mL [16.9–26.2]; combined remission: 21.6 µg/mL [17.1–24.8]).

### Predictive factors associated with response

Treatment with any biologic agent different to VDZ (anti-TNF, ustekinumab or JAK inhibitors) prior to surgery, was significantly associated with higher mPDAI score at week 12 and higher FC level at week 52 (p=0.045 and p=0.05, respectively). However, prior exposure to vedolizumab before IPAA did not significantly influence clinical or biochemical response (p=0,72 for mPDAI and p= 0,25 for FC level at week 52).

Post-surgical initiation of a first-line biologic therapy, other than VDZ, was associated with worse outcomes during follow-up; including higher mPDAI scores at week 12 (p = 0.031), elevated CRP levels at week 24 (p = 0.050) and increased FC levels at week 52 (p = 0.040).

Similar trends were observed in the subgroup of patients treated with SC VDZ. In this group, prior use of a non-VDZ first-line biologic therapy post-surgery was significantly associated with higher mPDAI score at week 52 (p < 0.001). However, no significant differences in FC or CRP levels were detected within this subgroup.

Concomitant corticosteroid and immunosuppressive therapy used during IV VDZ therapy did not significantly impact clinical outcomes.

### Vedolizumab persistence and discontinuation

**Figure 5:**
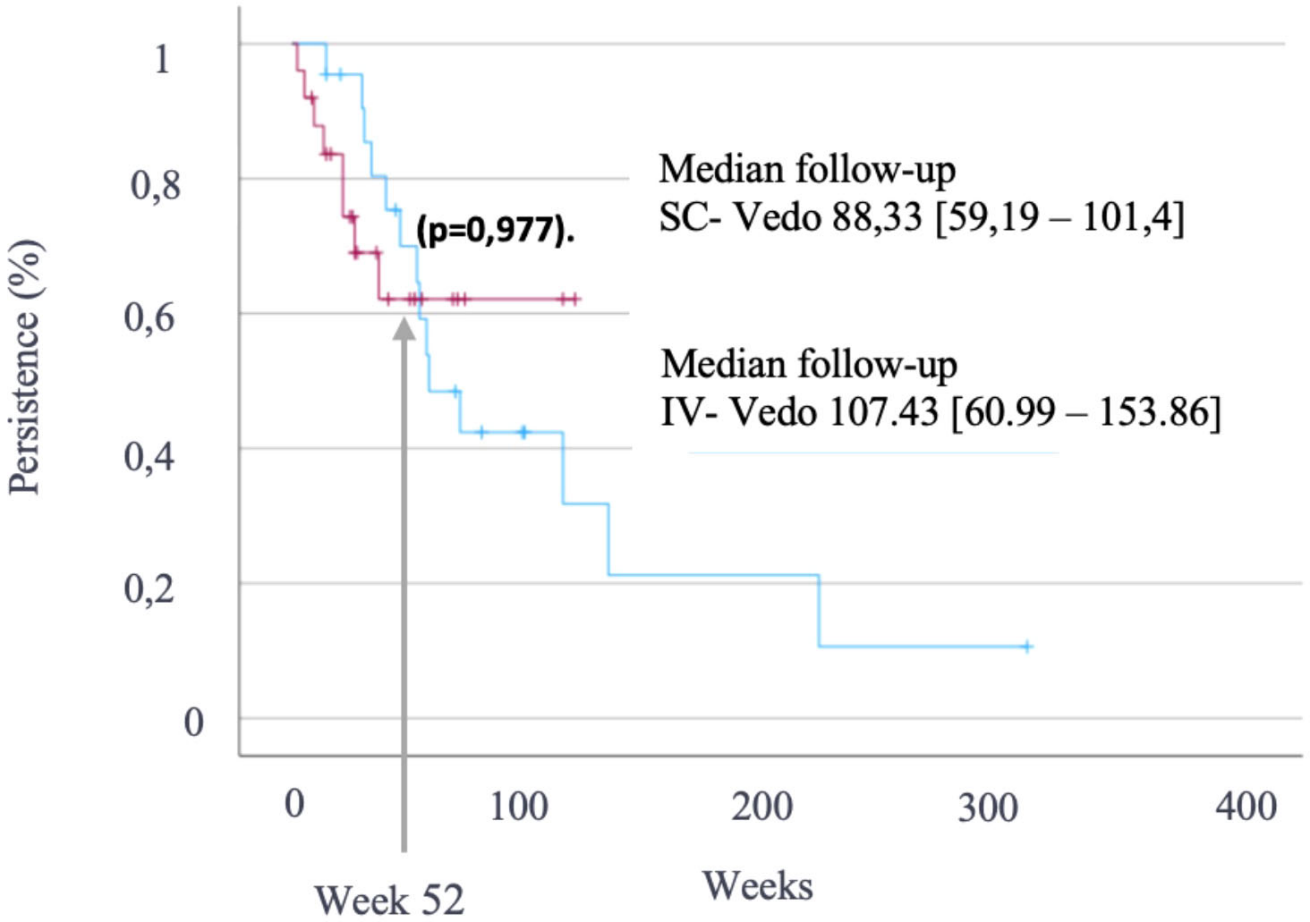
Kaplan–Meier curves showing vedolizumab persistence at 52 weeks in patients treated with intravenous vedolizumab (blue) and those who switched to subcutaneous administration (purple). Comparison by log-rank test (*p* = 0.977).

The mean follow-up duration was 107.4 weeks (95% CI: 61–153) for IV VDZ and 88.3 weeks (95% CI: 59.2–101.5) for SC VDZ. After confirming baseline comparability between both groups, VDZ persistence at 52 weeks was similar—68% for IV and 62% for SC administration—with no statistically significant difference (p = 0.977, log-rank test) (**Figure 5**).

Treatment discontinuation occurred in six patients receiving IV VDZ: four due to primary non-response and two due to secondary loss of response. Similarly, SC VDZ was discontinued in five patients: four due to loss of response and one due to an adverse event.

### Safety profile

The incidence of adverse events was similar between the IV and SC VDZ groups, reinforcing the favorable safety profile of VDZ in this real-world setting. A total of seven patients (15%) reported adverse events. In the IV group, reported events included two cases of pharyngitis, one case of fistulas, and one case of aphthous ulcers with associated arthralgia. In the SC group, three patients developed arthralgia. All adverse events were graded as mild to moderate in severity and none were classified as serious. Relatedness was judged by the treating physician: two cases of arthralgia and one case of pharyngitis were considered possibly related to VDZ, while the rest were considered unrelated. No malignancies or opportunistic infections occurred, and only one patient discontinued SC therapy due to an adverse event.

Regarding hospitalizations, 6 patients (12.8%) in the IV group required admission, with 3 undergoing surgeries (permanent ileostomy). In comparison, only one patient (4%) in the SC group was hospitalized and required surgery due to adhesive intestinal obstruction (**Table 5**).

**Table 5:** Incidence of adverse events, hospitalizations, and reasons for vedolizumab discontinuation in patients treated only with intravenous vedolizumab (n=22), as well as in those who transitioned from intravenous to subcutaneous administration (n=25).

	IV vedolizumab (N = 22)	SC vedolizumab (N = 25)
Adverse events	4 (8.5%)	3 (12%)
Hospitalizations	6 (12.8%)	1 (4%)
Surgery	3 (6.3%)	1 (4%)
Primary non-response	4 (8.5%)	0 (0%)
Loss of response	0 (0%)	6 (24%)

## Discussion

This is the largest prospective, multicenter real-world study with ≥52 weeks of follow-up evaluating both IV and SC VDZ in chronic pouchitis. Our results show significant improvements in disease activity and inflammation markers, with no loss of response after switching to SC VDZ. Instead, disease control was sustained or improved, supported by higher drug levels, maintained remission, and a favorable safety profile.

Long-term persistence of advanced treatment in patients with CARP is low. Ghersin et al. reported a maintenance rate in CARP patients on anti-TNF drugs of 22.4%, with a median time to discontinuation of 12.2 months (range 5.1–26.9 months). The main reason for discontinuation was lack of efficacy despite adequate levels and the absence of anti-drug antibodies (30.6%) (7). VDZ has been shown to be an effective and well-tolerated treatment option for chronic pouchitis, with clinical response rates around 60–70% and sustained benefit over time, even in patients previously exposed to anti-TNF therapy (11).

Our findings align with those reported in recent real-world studies evaluating the transition from IV to SC VDZ in IBD patients (19, 20). Notably, Wiken et al. observed 74% SC VDZ persistence at 18 months and highlighted that those patients in clinical remission at switch had significantly lower discontinuation rates (19). Like our results, other study observed a significant increase in serum trough concentrations following the switch to SC administration, without compromising clinical or biochemical outcomes (20). Although their cohort included patients with CD and UC rather than CARP, both studies reinforce the feasibility and safety of transitioning to SC VDZ in a real-life setting. Importantly, our study extends these findings by demonstrating that SC VDZ maintains disease control even in patients with complex post-surgical phenotypes such as CARP, a subgroup typically excluded from clinical trials.

One of the most notable aspects of our study is the real-world setting, where treatment decisions were driven by clinical practice rather than standardized protocols, enhancing the applicability of our findings. The relapsing and multifactorial nature of CARP often complicates treatment. Previous studies on anti-TNF agents had shown conflicting results, particularly in patients with prior exposure before colectomy (21–24).

In our cohort, approximately half of the patients had received biologic therapy prior to surgery, most commonly anti-TNF agents, while 14.9% had been previously treated with VDZ. This finding can be explained by the fact that, in our cohort, 36% of patients (n=16) underwent surgery before the approval of infliximab for UC in 2006, and 72% (n=32) underwent surgery before the approval of VDZ in 2014. Importantly, prior exposure to biologics appeared to negatively impact the subsequent clinical response to VDZ after IPAA.

It is well established that VDZ shows greater efficacy when used as first-line therapy in patients with moderate to severe UC, whereas its effectiveness diminishes in later lines of treatment (25, 26). Similarly, recent data from the REPREVIO trial demonstrated that VDZ is an attractive option for postoperative management in CD patients with high risk of recurrence (27). However, this phenomenon has not previously been described in the post-surgical setting in UC.

Recent studies have shown that vedolizumab tissue concentrations correlate with mucosal healing (28) and that vedolizumab may restore epithelial barrier function (29). Moreover, mucosal immune composition appears to modulate treatment response (30, 31). These findings support the hypothesis that a relatively preserved mucosal environment enhances vedolizumab efficacy. Our study provides novel evidence supporting the idea that first line use of VDZ after colectomy, specifically as the first-line biologic therapy, may lead to more favorable clinical and biochemical outcomes in patients with CARP. In fact, at treatment start, the majority of patients had mild pouchitis (median mPDAI 5), consistent with early therapeutic intervention rather than treatment of severe, relapsing disease. Conversely, patients who received other biologics prior to VDZ initiation exhibited worse response. This may be explained by the immunological context in which VDZ acts: VDZ has low immunogenicity, and its mechanism of action depends on a relatively preserved mucosal immune environment and epithelial barrier integrity, conditions that are more likely to be present in early stages of disease. In contrast, patients with long-standing or refractory UC often have chronic inflammation, structural tissue damage, and impaired barrier function, all of which may limit the therapeutic potential of VDZ (32).

Therapeutic drug monitoring (TDM) has become a valuable strategy in the management of IBD, aiming to optimize treatment efficacy and achieve objective, rigorous clinical endpoints. In IBD patients, non-operated UC, optimal VDZ serum concentrations vary depending on the therapeutic target, disease phenotype, inflammatory burden, and timing of sampling within the treatment cycle (33, 34). Higher VDZ levels during induction (typically >18 µg/mL) have been associated with improved rates of clinical remission, mucosal healing, greater treatment persistence, and a reduced need for dose intensification (35, 36). However, during maintenance phase, the correlation between VDZ levels and clinical outcomes is less clearly defined (36). It is also well established that switching from IV to SC VDZ leads to significantly increased drug levels, while maintaining efficacy, safety, and tolerability. Nevertheless, the optimal therapeutic concentration following this switch remains to be determined (37, 38).

In contrast to non-operated UC, data on TDM and pharmacokinetics in patients with CARP are scarce. To our knowledge, this is the first study to describe serum VDZ levels in this population and to evaluate the impact of switching from IV to SC administration in this specific clinical context.

In our cohort, SC VDZ trough levels in patients who achieved remission remained consistently high and stable across all timepoints, due the low immunogenicity of VDZ, suggesting that SC administration may offer a more predictable and sustained pharmacokinetic profile for maintaining clinical remission in CARP. Conversely, IV VDZ levels were more variable and, paradoxically, tended to be higher in non-remitters at later timepoints (notably at weeks 24 and 52), potentially reflecting reactive intensification strategies and further questioning the long-term predictive value of higher IV drug levels similarly to UC non-operated patients.

Moreover, achieving combined clinical and biochemical remission was associated with numerically higher VDZ levels than clinical remission alone, particularly in the SC group. These findings support the hypothesis that SC VDZ may provide a more reliable exposure–response relationship and could be especially advantageous for reaching ambitious therapeutic goals in patients with CARP.

VDZ persistence at 52 weeks was high in our study, 68% for IV and 62% for SC administration, probably due to its low immunogenicity. In this sense, Verstockt et al. reported significantly greater long-term treatment durability with VDZ compared with anti-TNF agents (HR 3.0; 95% CI 1.1–8.7; p = 0.04), with more patients discontinuing anti-TNF therapy as early as week 14 (39).

Adverse event rates were low in our study compared to those reported with anti-TNF therapy, where infusion reactions accounted for 40.7% of treatment discontinuations (34). We found a comparable rate of adverse events between IV (8.5%) and SC (12%) formulations, supporting the safety of both VDZ and the SC administration in CARP patients.

While our findings are encouraging, several limitations must be acknowledged. Being an observational multicenter study, data collection may vary slightly across centers. Moreover, treatment initiation and switching to SC VDZ were not protocol-driven but they were based on clinical judgment, reflecting everyday practice. Endoscopic data were available in only ~60% of cases during follow-up, limiting objective assessment in some patients. In these cases, disease activity was evaluated clinically, which may have affected the interpretation of VDZ effectiveness. Concomitant pre-pouch ileitis (PPI), as an inflammation of the ileum immediately proximal to the pouch, without necessarily meeting the criteria for Crohn’s disease, is a similar clinical sub-condition which development seems to be an extension of the pouchitis or a non-specific inflammatory response of the adapted ileum. Definitive diagnosis is histological and its presence can influence the clinical and endoscopic response of patients with CARP (40). Nevertheless, Ghersin et al. recently demonstrated similar results regarding the efficacy and persistence of anti-TNF drugs as first-line options in patients with concomitant PPI (41). Given the limited sample size, mixed-effects modelling and landmark analysis were not feasible in this study.

In conclusion, this real-world study is the first to evaluate the pharmacokinetics and outcomes of VDZ in chronic pouchitis following a switch from IV to SC administration. Our findings support VDZ as an effective and safe treatment for chronic pouchitis, particularly when used as first-line therapy after IPAA. The SC formulation maintains long-term remission and may help reduce the risk of pouch failure. Further studies are needed to validate these results.

## Data Availability

The raw data supporting the conclusions of this article will be made available by the authors, without undue reservation.
